# Shared Human/Rabbit Ligands for Rabbit Hemorrhagic Disease Virus

**DOI:** 10.3201/eid1803.111402

**Published:** 2012-03

**Authors:** Kristina Nyström, Béatrice Le Moullac-Vaidye, Nathalie Ruvoën-Clouet, Jacques Le Pendu

**Affiliations:** Institut National de la Santé et de la Recherche Médicale, Nantes, France;; University of Nantes, Nantes

**Keywords:** Calicivirus, viruses, rabbit hemorrhagic disease virus, attachment factors, human/rabbit ligands, histo-blood group antigen, ABO, secretor, host-switching

**To the Editor:** Rabbit hemorrhagic disease virus (RHDV) is a calicivirus of the genus *Lagovirus* that causes epidemics of an acute disease and mortality rates of 50%–90% among rabbits. The disease, which was first described in 1984, is characterized by hemorrhagic lesions, mainly affecting the liver and lungs 24–72 h after infection (*1*).

Similar to human caliciviruses of the genus *Norovirus*, RHDV binds to histo-blood group antigens (HBGAs), and we recently showed that HBGAs serve as attachment factors (ligands) that facilitate RHDV infection (*2*). HBGAs are polymorphic carbohydrate structures representing terminally exposed portions of larger glycans linked to proteins or glycolipids. In many vertebrate species, they are mainly expressed on epithelial surfaces. Because phylogenetic conservation of receptors is a major risk factor for cross-species transmission (*3*), we analyzed the ability of RHDV strains to recognize human HBGAs expressed on epithelia.

We obtained 38 saliva samples from healthy persons with ABO, Secretor, and Lewis phenotypes, and we selected confirmed *FUT2* (secretor) and *FUT3* (Lewis) genotypes (*4*) to include ABO, secretor, and Lewis phenotypic diversity. Binding capacity of 6 RHDV strains representative of virus diversity (*2*) was tested against human saliva samples by using a method similar to that reported for human norovirus (*5*).

In brief, saliva samples diluted 1:1,000 or B type 2 bovine serum albumin–conjugated tetrasaccharide (positive control) were coated on ELISA plates. After blocking with milk diluted in phosphate-buffered saline, RHDV strains isolated from whole liver extracts of infected animals were incubated on coated plates at dilutions corresponding to 1 × 10^9^ genome copies (0.2 µg/mL capsid protein equivalent) as determined by Nyström et al. (*2*). Monoclonal antibody 2G3, biotinylated anti-mouse IgG, and peroxidase-conjugated avidin were used for RHDV detection; 3,3′,5,5′-tetramethylbenzidine was used as a substrate; and optical density values at 450 nm were measured (*2*).

Binding to the B type 2 epitope was observed for all 6 strains (Technical Appendix Figure, panel A). Human saliva samples were recognized by 5 of 6 RHDV strains. Only G6, an RHDV antigenic variant also known as RHDVa (*6*), did not show binding to saliva. Strains G1 and G2 showed preferential binding to saliva from B secretors over that from O secretors, and A secretors were poorly recognized. Better recognition of A secretor saliva was obtained with the G3 strain. The G4 and G5 strains showed a clear preference for A secretors over B and O secretors, which indicated a shift in specificity toward recognition of the A antigen from the H and B antigens, as reported (*2*). None of the strains recognized nonsecretor saliva, which showed that binding to human saliva required A, B, or H motifs. This finding was confirmed by drastically decreased binding after removal of A, B, and H epitopes from secretor saliva by treatment with specific glycosidases. There was no relationship with the Lewis status.

To determine if human epithelial cells were recognized by RHDV, binding of the G3 strain to human tissue sections was assessed. Human trachea, lung, and gastroduodenal junction samples obtained from organ donors (before current French restrictions of December 1988) were used to prepare tissue microarrays. Tissues from 18 persons were used and represented the following phenotypes: O secretor Lewis+ (n = 8), A secretor Lewis+ (n = 3), B secretor Lewis+ (n = 2), O secretor Lewis– (n = 1), and O nonsecretor Lewis+ (n = 4). Deparafinated and endogen peroxidase–blocked sections were incubated overnight at 4°C with the G3 strain from an infected liver extract at a concentration of 2 × 10^9^ genome copies/mL.

Binding was detected by using monoclonal antibody 2G3 against RHDV, biotinylated anti-mouse IgG, horseradish peroxidase–conjugated avidin, and 3-amino-9-ethylcarbazole substrate with hemalum counterstaining, as described (*2*). Staining of epithelial cells of stomach or trachea of secretors, but not those of nonsecretors, was observed. This finding indicated that attachment factors for RHDV are present on human cells that constitute potential points of entry for RHDV (Technical Appendix Figure, panels B–E).

Attachment to HBGAs of human calicivirus strains represents a first step of the infection process (*7*). In this study, we have shown that cross-species recognition of HBGAs in cells that may be likely points of entry of RHDV into human cells. RHDV infection has been shown to be rabbit specific (*8*), which indicates that other molecular elements not shared by rabbits and other mammals restrict its host range. Nevertheless, RHDV RNA was recently isolated from sympatric wild small mammals, which suggested that the species range of RHDV may not be as limited as previously believed (*9*). In addition, recent phylogenetic analysis showed that caliciviruses exhibit high levels of host switching (*10*). Therefore, surveillance of RHDV and studies to decipher molecular mechanisms involved in its extreme pathogenicity are warranted.

## Supplementary Material

Technical Appendix Binding of rabbit hemorrhagic disease virus (RHDV) to human saliva samples.
