# Supplementary material for: Shared Human/Rabbit Ligands for Rabbit Hemorrhagic Disease Virus
Source: Emerg Infect Dis. 2012 Mar;18(3):518–9. doi: 10.3201/eid1803.111402 (PMC3309641; doi:10.3201/eid1803.111402)
Supplement: Technical Appendix — Binding of rabbit hemorrhagic disease virus (RHDV) to human saliva samples. [file 11-1402-Techapp_1p.pdf]

Article DOI: http://dx.doi.org/10.3201/eid1803.111402

Shared Human/Rabbit Ligands for Rabbit Hemorrhagic Disease Virus

Technical Appendix

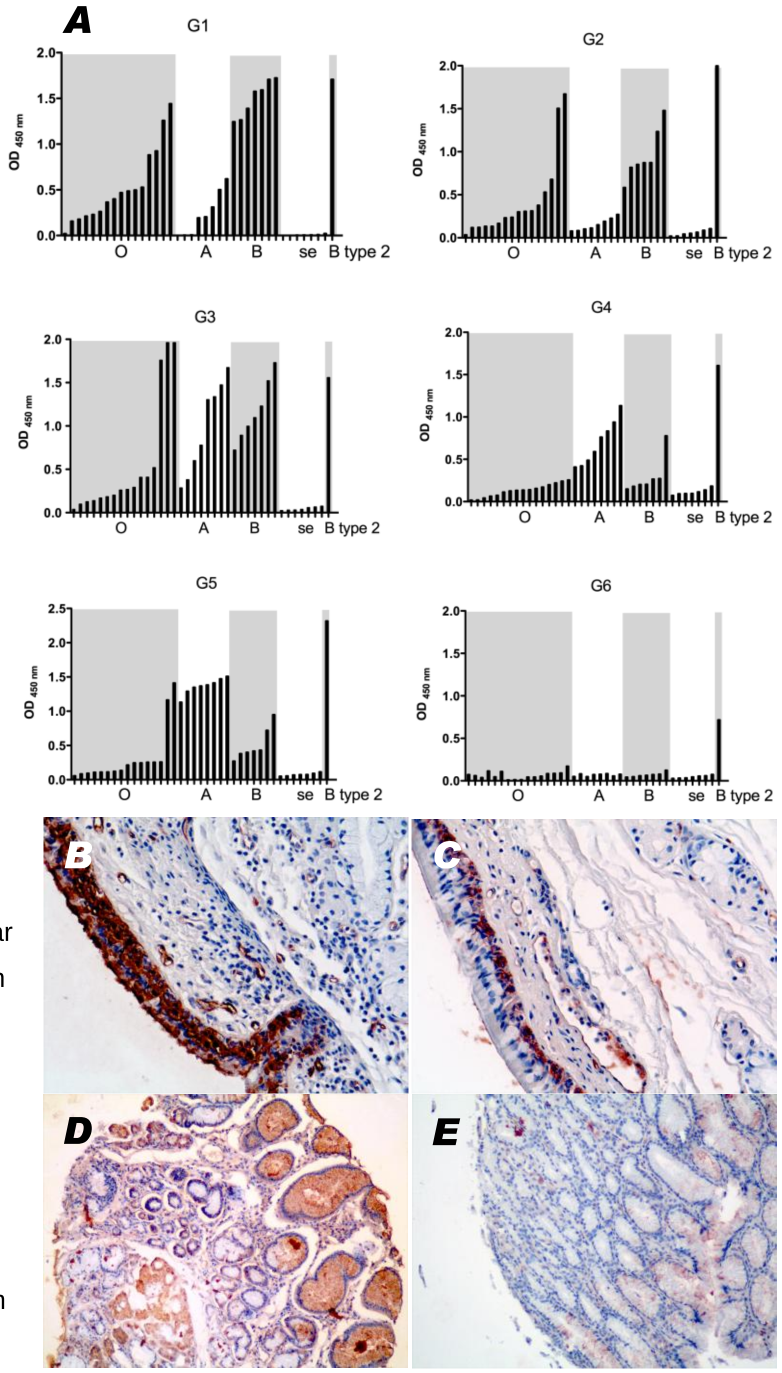

Technical Appendix Figure. Binding of rabbit hemorrhagic disease virus (RHDV) to human saliva samples. A) Histo-blood group antigens of saliva samples are shown at the bottom of each panel, corresponding to binding of strains G1–G6. O, A, and B, secretors (separated by shading); se, nonsecretors. B type 2 represents synthetic B type 2 tetrasaccharide conjugated to bovine serum albumin and used as a positive control. Bars show optical density at 450 nm (OD_{450}). Signal above background values was not observed when whole liver extract from an uninfected animal was used. B–E) staining of human tissues for the G3 strain of RHDV. B) Trachea of an O secretor, showing staining of the entire epithelial layer and vascular endothelium in underlying connective tissue. C) Trachea of an O secretor, showing staining of only basal epithelial cells and vascular endothelium. D) Pyloric area of gastroduodenum junction of an O secretor, showing staining of surface epithelium. E) Pyloric area of an O nonsecretor, showing absence of staining of surface epithelium (monoclonal antibody 2G3 against RHDV, biotinylated anti-mouse IgG, horseradish peroxidase–conjugated avidin, and 3-amino-9-ethylcarbazole–stained with hemalum [hematoxylin and alum] counterstain.) Original magnifications ×400 in B and C and ×100 in D and E.
